# Effects of the different Tai Chi exercise cycles on patients with essential hypertension: A systematic review and meta-analysis

**DOI:** 10.3389/fcvm.2023.1016629

**Published:** 2023-03-03

**Authors:** Yikun Yin, Zhengze Yu, Jialin Wang, Junzhi Sun

**Affiliations:** ^1^ College of Physical Education and Health, Guangxi Normal University, Guilin, China; ^2^ Institute of Sports Medicine and Health, Chengdu Sport University, Chengdu, China

**Keywords:** Tai Chi, essential hypertension, exercise cycle, blood lipid metabolism, meta-analysis

## Abstract

**Objective:**

The main treatment for essential hypertension at this stage is pharmacotherapy. Long-term pharmacotherapy is costly with some side effects. Tai Chi, a bright star in traditional Chinese arts, relaxes both mind and body and has been shown to relax blood vessels and lower blood pressure. This study aimed to systematically review the therapeutic effectiveness of the Tai Chi exercise cycle on blood pressure and cardiovascular risk factors of patients with essential hypertension.

**Methods:**

Searching CNKI, VIP, CBM, PubMed, EBSCO, Embase, Cochrane Library, and Web of Science to collect randomized controlled trials about Tai Chi exercise in the treatment of patients with essential hypertension according to the inclusion and exclusion criteria. The search time ranged from the date of database construction to December 2022. The Cochrane risk-of-bias tool was used to evaluate the included trials. The meta-analysis was performed with RevMan5.3 and Stata12.0 software.

**Results:**

According to the meta-analysis, compared with the controls, Tai Chi exercise with a cycle of more than 12 weeks may be better for the reduction of systolic blood pressure (SBP) [MD = −11.72, 95% CI (−15.52, −7.91)] and diastolic blood pressure (DBP) [MD = −4.68, 95% CI (−7.23, −2.12)], as well as increasing the content of nitric oxide (NO) [MD = 0.99, 95% CI (0.69, 1.28)]. The blood lipid metabolism ability may also be improved after more than 12 weeks of Tai Chi exercise, total cholesterol (TC) [SMD = −0.68, 95% CI (−0.89, −0.46), triglyceride (TG) [SMD = −0.84, 95% CI (−1.25, −0.43)], low-density lipoprotein cholesterol (LDL-C) [SMD = −1.58, 95% CI (−2.29, −0.86)]. However, the improvement of high-density lipoprotein cholesterol (HDL-C) [SMD = 0.54, 95% CI (0.28, 0.79)] was better with a less than 12 weeks exercise cycle. A subgroup analysis for exercise frequency and time showed that the exercise frequency should preferably be more than or equal to 5 times per week for patients with hypertension, and for patients with hypertension plus hyperlipidemia, the exercise frequency less than 5 times per week with exercise time less than 60 min each day may be more beneficial.

**Conclusion:**

The meta-analysis indicated that a more than 12 weeks Tai Chi exercise cycle with less than 60 min each time and more than 5 times per week may be more beneficial in blood pressure reduction, NO level increasing and blood lipid metabolism improving in the comparison with the other exercise cycles. For patients with hypertension plus hyperlipidemia, exercise frequency of less than 5 times per week may be better.

**Systematic Review Registration:**

[http://www.crd.york.ac.uk/prospero], identifier [CRD42022352035].

## 1. Introduction

Essential hypertension (EH) is a clinical syndrome characterized by elevated systemic arterial pressure (1). EH is not only the disease with the highest incidence, but it is also the major risk factor for heart and cerebrovascular disease. According to statistics, there are 270 million patients with EH in China (2019). The incidence rate of hypertension is expected to climb to 29% (2). At this stage, drug therapy is the main treatment for hypertension. However, the long-term expense of taking prescriptions is so high, and the side effects of the medications are so considerable that individuals with hypertension have poor compliance (3). Aerobics activities such as walking, jogging, and swimming for at least 30 min per day, 5 days per week may effectively reduce blood pressure in patients with hypertension and prehypertension (4–6). In addition to pharmacotherapy and physical exercise, changing unhealthy lifestyles is also an important approach for the prevention of cardiovascular disease (CVD), favoring the control of blood pressure, such as a low salt diet, reduced intake of red meat, sugar, and trans-fat, etc. (7–9). According to the World Health Organization 2020 guidelines on physical activity and sedentary behavior, physical activity can reduce cardiovascular disease mortality, postpone disease progression, improve bodily function, and improve the quality of life for individuals with hypertension (10).

Tai Chi is a prominent traditional Chinese martial art as well as a popular Chinese aerobic exercise. It's also recognized as a traditional type of rehabilitative training. Tai Chi is conducive to maintaining the stability of the vasomotor nerve, improving vascular compliance, reducing blood pressure, enhancing cardiorespiratory ability, and improving the quality of life (11). Compared with drug therapy, Tai Chi has fewer negative effects on patients with hypertension. Tai Chi encourages the production of nitric oxide, improves vasodilation, and lowers blood lipid levels in patients (12). Among numerous traditional Chinese methods, Tai Chi is the most effective one in improving the quality of life in patients with essential hypertension (13, 14). A meta-analysis of Tai Chi for EH patients reported significant reductions in SBP and DBP with an exercise cycle of 12–24 weeks (15). Guan et al. suggested that Tai Chi can reduce SBP and DBP while exercising cycles at both less than 12 weeks and more than or equal to 12 weeks (16). Through a collation of the available literature, we found that previous systematic reviews only made a rough summary of the effects of Tai Chi exercise cycles on blood pressure, and did not analyze blood lipid metabolic ability, as a relevant indicator of cardiovascular risk factors, of patients with hypertension. In addition, the time and frequency of each exercise were not described in detail, making it impossible to effectively formulate a reasonable exercise prescription.

In summary, considering the different influences on blood pressure, blood lipid, and serum NO levels in EH patients caused by the differences in Tai Chi exercise cycle, time, and frequency, we used meta-analysis to integrate the recent research results of Tai Chi exercise intervention in essential hypertension and carried out systematic, objective and quantitative statistical analysis. We conducted a systematic review and meta-analysis of randomized controlled trials (RCTs) of Tai Chi Exercise on hypertension in domestic and international databases to see whether the cycle of Tai Chi exercise has different effects on patients with essential hypertension, in order to provide more objective and scientific exercise prescriptions in the future.

## 2. Materials and methods

### 2.1. Retrieval strategy

This meta-analysis was planned and implemented according to the Preferred Reporting Items for Systematic Reviews and Meta-Analyses (PRISMA) guidelines (17). The protocol was registered on the international prospective register of systematic reviews (http://www.crd.york.ac.uk/PROSPERO) with a registration number CRD42022352035.

A search of CNKI, VIP, CBM, PubMed, EBSCO, Embase, Cochrane Library, and Web of Science for RCTs on Tai Chi exercise on patients with essential hypertension published from the time of the databases established to December 2022 was conducted on December 13, 2022. We also searched to retrieve all potential relevant unpublished reported materials and conference proceedings referred to the topic. Search terms included “tai-ji”, “tai chi”, “chi, tai”, “tai ji quan”, “ji quan, tai”, “quan, tai ji”, “taiji”, “taijiquan”, “t’ai chi”, “tai chi chuan”, “hypertension”, “blood pressure, high”, “high blood pressure”, “hypertension, essential”, “essential hypertension”, “primary hypertension”, “human essential hypertension”, “idiopathic hypertension”. The full search strategies of each database were presented in Supplementary Material S1.

### 2.2. Literature inclusion, exclusion criteria, and outcome indicator

Inclusion criteria: (1) The study design was a randomized controlled trial (RCT). (2) The research objects were patients with essential hypertension (unlimited sex, age, race, and nationality) according to the diagnostic criteria such as 1999/2005/2010/2016 Chinese guidelines for the management of hypertension, WHO-ISH (i.e., SBP ≥140 mmHg and/or DBP ≥90 mmHg) (18–23). Patients with secondary hypertension and other severe cardio-cerebrovascular disease were required to be excluded. (3) The main intervention methods were Tai Chi exercise or other intervention methods combined with Tai Chi exercise. The intervention methods of the controls included pharmacotherapy, usual care, other exercise methods, or no treatment. (4) Raw data were complete and could be extracted directly or indirectly for analysis. (5) The publication language of the articles was Chinese or English.

Exclusion criteria: (1) Duplicate published literature; (2) Inability to efficiently extract data and access the literature of original articles; (3) Animal studies or cross-sectional studies; (4) Experiments with nonclinical and nonintervention designs.

Outcome indicators included systolic blood pressure (SBP), diastolic blood pressure (DBP), total cholesterol (TC), triglycerides (TG), low-density lipoprotein cholesterol (LDL-C), high-density lipoprotein cholesterol (HDL-C) and serum nitric oxide (NO).

The abnormality of TC, TG, LDL-C, and HDL-C can induce or aggravate hypertension. The abnormality of blood lipids may lead to hypertrophy of smooth muscle cells on the vascular wall and deposition of collagen, thus causing structural changes in the large artery vessels. At the same time, the damage of renal microvessels caused by dyslipidemia is also one of the causes of hypertension.

### 2.3. Literature screening and information extraction

Step 1: Import retrieved literature to the literature management software Endnote X9 (www.endnote.com). Step 2: Exclude duplicate materials. Step 3: Perform the first round of screening by reading titles and abstracts. Step 4: After downloading full texts, conduct the second round of screening to determine if the inclusion criteria were met.

Two independent reviewers, ZY and YY, conducted the literature screening and data extraction. Then cross-checking was performed. When a possible disagreement occurred, we solved it through discussion or negotiation with a third independent reviewer, JW. In literature screening, we first read the title to exclude irrelevant literature. And then, we further read the abstract and the full text to determine whether to include it. If necessary, we would contact the author of the original research by email or telephone to obtain the unconfirmed information. The extraction data included general information about the included literature (the title, the first author, and the year of publication), general characteristics of the patients (the number of cases in each group, the age, and the duration of the disease), treatment specifics and the follow-up time, key elements of bias risk assessment, and focused outcome indicators.

### 2.4. Quality assessment

Two independent reviewers, ZY and YY, used the Cochrane Collaboration tool to examine the risk of bias for the included studies (24, 25), and cross-checking was conducted. A literature quality grade was performed according to the Jadad Scale. A score of 1–3 was considered low quality, and a score of 4–7 was considered high quality. The grading was also conducted by two independent reviewers, with the disagreement consulting the opinions of a third independent reviewer, JW.

### 2.5. Statistical analysis

The statistical analysis was based on RevMan5.4 (the Review Manager software 5.4, The Nordic Cochrane Center, The Cochrane Collaboration). If the results included in the literature were continuous variables and from the same assessment method, use the mean difference (MD) and 95% confidence interval (CI) for statistics. If the results were not from the same assessment method, the standard mean difference (SMD) and 95% confidence interval (CI) were conducted. The *p*-value and the *I*^2^ index were used as indicators to assess the heterogeneity among studies. There was no heterogeneity between studies when *p* ≥ 0.10, while *p* < 0.10 indicates that there was heterogeneity between studies. The *I*^2^ index represented the degree of heterogeneity between studies. If *I*^2^ < 50%, it indicates that there was slight heterogeneity between the studies, and the fixed effect model was used for analysis. If *I*^2^ ≥ 50%, there was heterogeneity in the study, and the random effect model was used for analysis (26). The *α* value was set at 0.05. And Stata 12.0 software was used to conduct the publication bias analysis and sensitivity analysis of Begg's test for the studies with more than 5 included outcome indicators. The threshold for statistical significance was set at *p* < 0.05.

## 3. Results

### 3.1. Study selection

**Figure 1:**
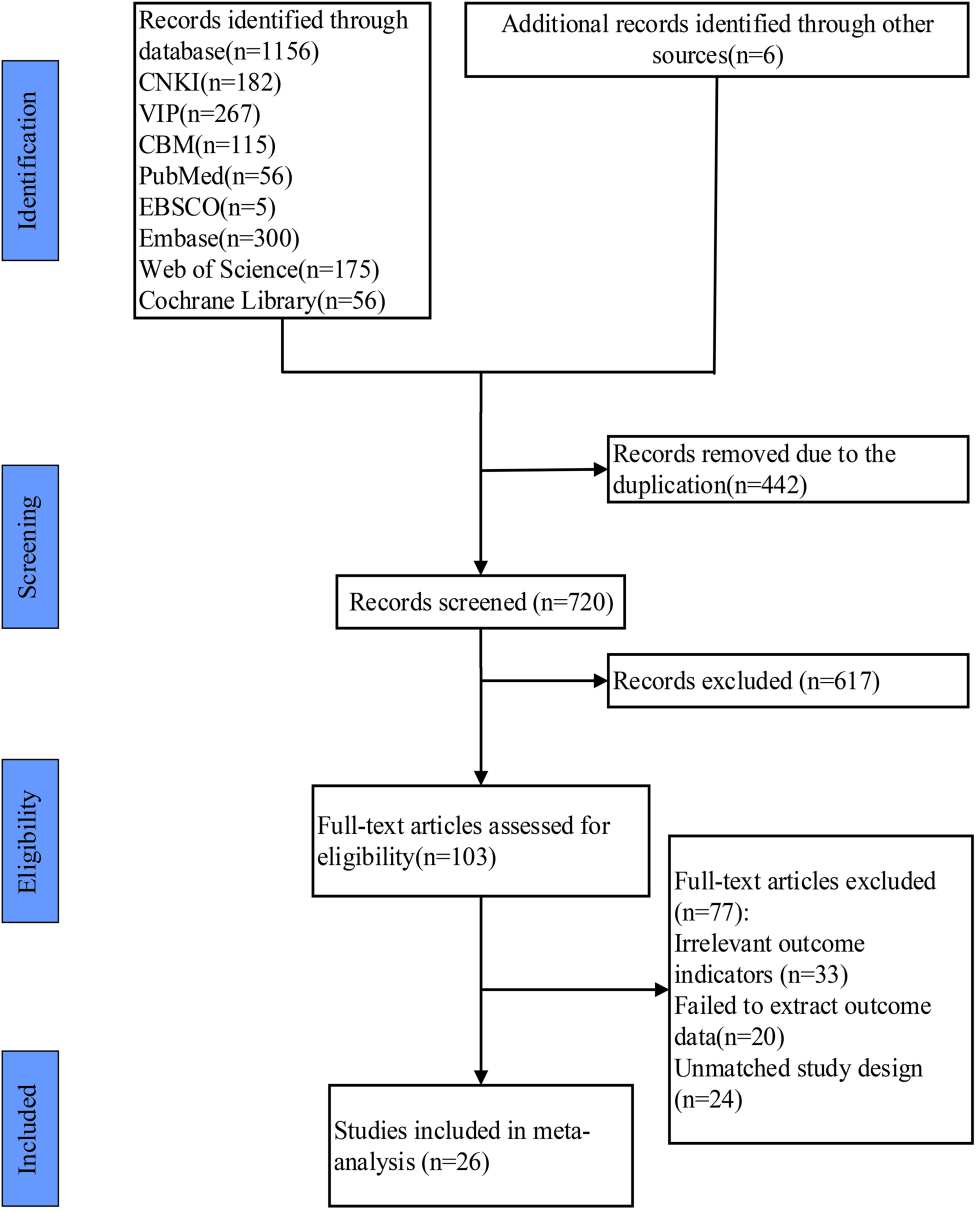
Study selection represented by PRISMA flowchart.

Through the database search, 1,162 articles were preliminarily collected and 6 articles were obtained through a retrospective review of the references. Then we eliminated duplicate publications, 720 articles were left. Determining the research content by reading the titles and abstracts, we screened out 103 articles. After reading the full text, we finally selected 26 articles (23, 27–51) according to the inclusion and exclusion criteria. The literature screening process and results were shown in Figure 1.

### 3.2. Assessment of publication bias

**Figure 2:**
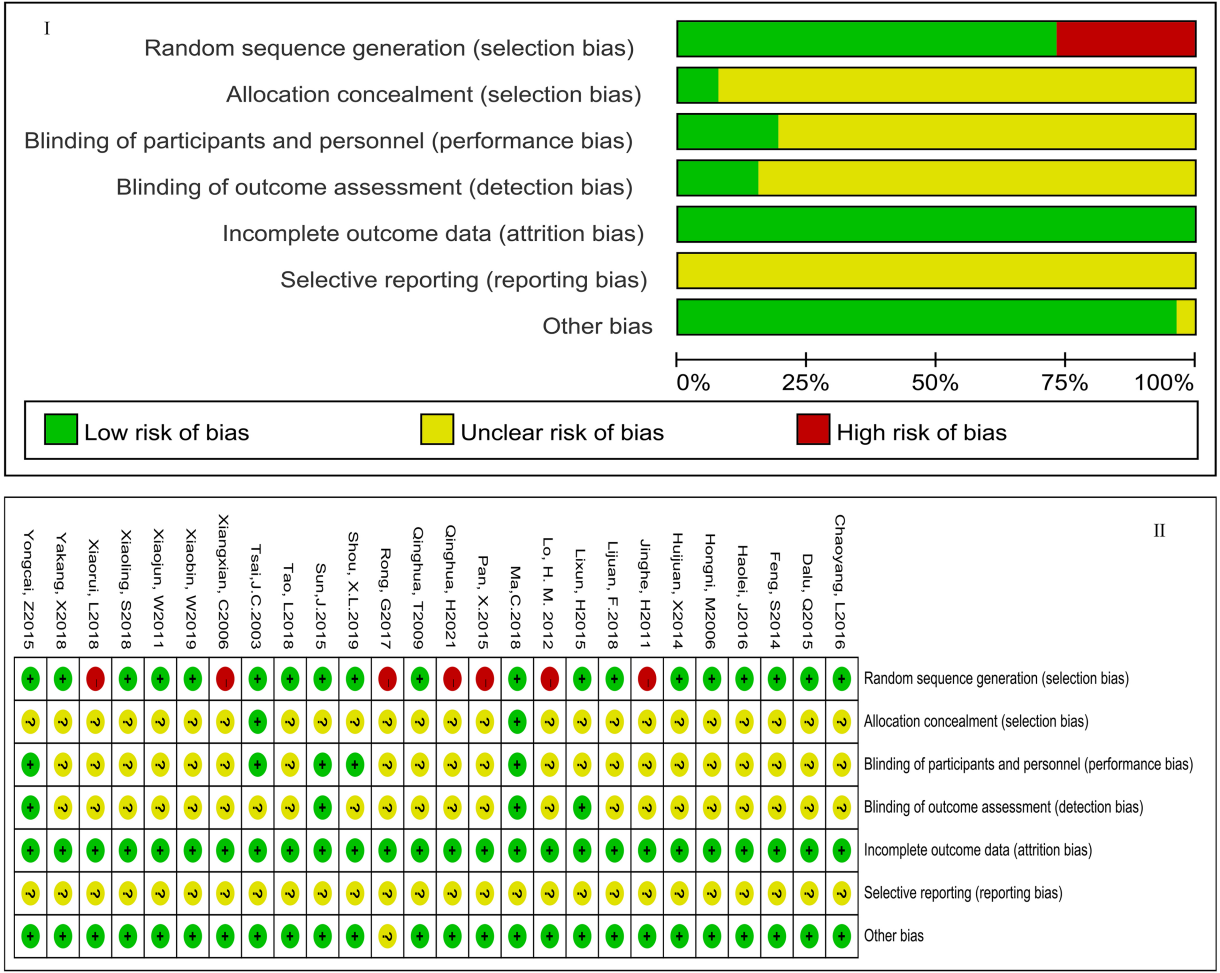
The risk of publication bias. Figure 1 showed the overall risk of bias in the included studies. Figure 2 showed the specific risk of bias in each study. The green part meant low-risk bias, the red part meant high-risk bias, and the yellow part meant that risk bias was not clear; “+” meant low-risk bias; “−” meant low-risk bias; “?” meant risk bias is not clear.

The results of the risk assessment are shown in Figure 2. According to the Jadad scale, 5 articles were judged to be of low quality, and the remaining articles were considered to be of high quality. The detailed information was presented in Supplementary Material S2.

### 3.3. Basic characteristics of the article

According to the inclusion and exclusion criteria, a total of 26 articles were finally included, with a total of 2,370 participants. The exercise cycle ranged from 5 weeks to 1 year. In most studies, the intervention method of experimental groups was Tai Chi exercise alone. In a small number of studies, the intervention methods of experimental groups were Tai Chi exercise combined with pharmacotherapy, aerobic exercise, usual care, walking, or the Numan health care system. For the controls, the intervention method in most included studies was no treatment. And in the remaining studies, the method was the way after the removal of Tai Chi exercise. The characteristics and the main results of the studies were presented in Table 1.

**Table 1:** The details of research characteristics.

Author and year of publication	Year	Sample size (E/C)	Diagnostic criteria	Intervention (E)	Intervention (C)	Frequency/week	Exercise time/day	Exercise cycle	Outcome indicators	Follow-up
(27)	35–65	39/37	–	Tai Chi	No exercise intervention	3/W	50 min	12 w	①②④⑤⑥⑦	yes
(28)	45–70/52–72	51/11	GMH-1999	Tai Chi	No exercise intervention	6/w	60 min	8 w	①②③⑨	no
(29)	64.3/60.7	20/20	CGMH-2005	Tai Chi + nifedipine	nifedipine	7/W	40 min	10 w	①②③	no
(30)	63.65 ± 8.71/62·79 ± 7·43	16/16	WHO-ISH	Tai Chi	hypotensor	3–5/W	30–60 min	6 m	①②	no
(31)	51.6 ± 5.3/49.6 ± 7.5	33/16	CGMH-2009	Tai Chi	No exercise intervention	6/w	40–50 min	20 w	①②④⑤⑧	no
(32)	50–70	30/30	–	Tai Chi	No exercise intervention	5/W	45 min	16 w	①②	no
(33)	58.47 ± 7.46	27/31	–	Tai Chi	Conventional treatment	3/W	60 min	8 w	①②	no
(34)	68.16 ± 4.43 /69.10 ± 4.28	38/42	CGMH-2010	Tai Chi	Conventional treatment	7/W	120 min	8 w	①②	no
(35)	60–70	25/25	CGMH-2005	Tai Chi	No exercise intervention	5/W	60 min	12 w	①②③	no
(36)	52.62–68.74	30/30	CGMH-2005	Tai Chi	No exercise intervention	5/W	60 min	12 w	①②	no
(37)	64.10 ± 7.03/64.21 ± 6.12	55/55	–	Tai Chi + Walking	No exercise intervention	5/W	45 min	3 m	①②④⑤⑥⑦ ⑧	no
(38)	56.371 ± 3.95 /56.88 ± 3.95	24/16	CGMH-2010	Tai Chi	No exercise intervention	6/W	60 min	12 w	①②③④⑤⑥⑦⑧	no
(39)	45–80	136/130	–	Tai Chi	No exercise intervention	5/W	60 min	12 m	①②④⑤⑥⑦⑧	no
(40)	54.71 ± 5.43/55.77 ± 6.24	49/49	CGMH-2010	Tai Chi + nifedipine	nifedipine	4–8/W	40–60 min	12 w	①②④⑤⑥⑦	no
(41)	62.0 ± 2.2	60/60	CGMH-2010	Tai Chi	captopril	14/W	80 min	2 m	①②	no
(42)	56.9 ± 5.7	27/27	CGMH-2010	Tai Chi	Amlodipine	7/W	40 min	6 w	①②③⑨	no
(43)	75.38 ± 5.69/74.29 ± 4.58	54/54	–	Tai Chi + NuMan system	Conventional treatment	–	–	6 m	①②	no
(23)	60–80	41/41	CGMH-2016	Tai Chi	Walking exercise	3/W	60 min	12 w	①②④⑤⑥⑦	no
(44)	70.24 ± 10.25/ 69.71 ± 10.84	55/58	–	Tai Chi	Conventional treatment	3–5/W	60 min	24 w	①②	no
(45)	62.4 ± 2.4/63.1 ± 2.1	35/35	CGMH-2010	Tai Chi + cilazapril	cilazapril	7/W	40–60 min	24 w	①②	yes
(46)	52.35 ± 3.26/51.35 ± 4.21	98/100	CGMH-2010	Tai Chi	No exercise intervention	7–14/W	45–60 min	12 w	①②	no
(47)	51.08 ± 8.77/50.51 ± 8.68	61/61	–	Tai Chi	Conventional treatment	5–8/W	50–60 min	12 w	①②	no
(48)	60.2 ± 4.6/60.5 ± 4.9	42/42	CGMH-2010	Tai Chi + aerobic exercise	aerobic exercise	5/W	50 min	12 w	①②③⑧⑨	no
(49)	51.5 ± 6.78 51.5 ± 8.26	104/104	CGMH-2010	Tai Chi	No exercise intervention	6/W	40–90 min	3 m	①②④⑤⑥⑦	no
(50)	67.6 ± 4.5/67.4 ± 4.2	50/50	CGMH-2010	Tai Chi	Conventional treatment	3/W	40–60 min	12 w	①②④⑤⑥⑦⑧	yes
(51)	54 ± 6/53 ± 8	15/15	–	Tai Chi	No exercise intervention	3/W (1–6 W)	40–60 min	12 w	① ② ③	no

E, Experimental group; C, Control group; w, week; m, month; y, year; ①, Systolic blood pressure (SBP);②, Diastolic blood pressure (DBP); ③, Nitric oxide (NO); ④, Total cholesterol (TC); ⑤, Triglycerides (TG); ⑥, Low-density lipoprotein cholesterol (LDL-C); ⑦, High-density lipoprotein cholesterol (HDL-C); ⑧, Blood glucose; ⑨, Vascular endothelin “–”, not mentioned.; d, day; m, month; y, year.

### 3.4. Meta-analysis results

#### 3.4.1. The effect of Tai Chi exercise cycle on blood pressure

**Figure 3:**
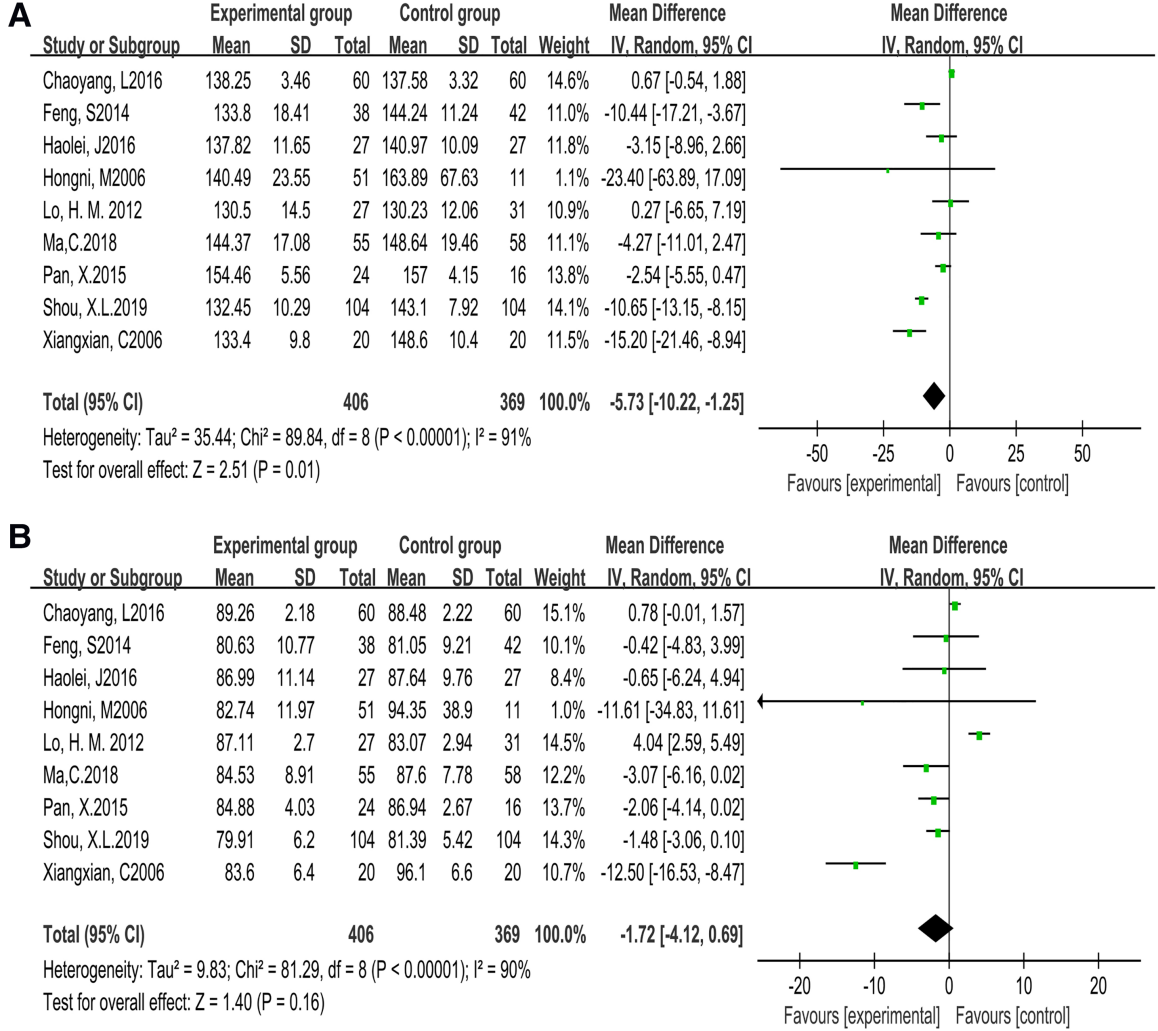
Forest plot of meta-analysis on the effect of Tai Chi exercise cycle on blood pressure (cycle <12 weeks). **A**, systolic blood pressure (SBP); **B**, diastolic blood pressure (DBP).

Based on 9 studies (775 participants), we found there was heterogeneity in systolic blood pressure (*P* < 0.00001, *I*^2^ = 91%) [MD = −5.73, 95% CI (−10.22, −1.25), *P* = 0.01] and diastolic blood pressure (*P* < 0.00001, *I*^2^ = 90%) [MD = −1.72, 95% CI (−4.12, 0.69), *P* = 0.16] while the cycle was less than 12 weeks, which was analyzed by random effects model. The result was not statistically significant, which meant that compared with the control group, Tai Chi exercise failed to effectively reduce the systolic and diastolic blood pressure levels. The results were displayed in Figure 3.

**Figure 4:**
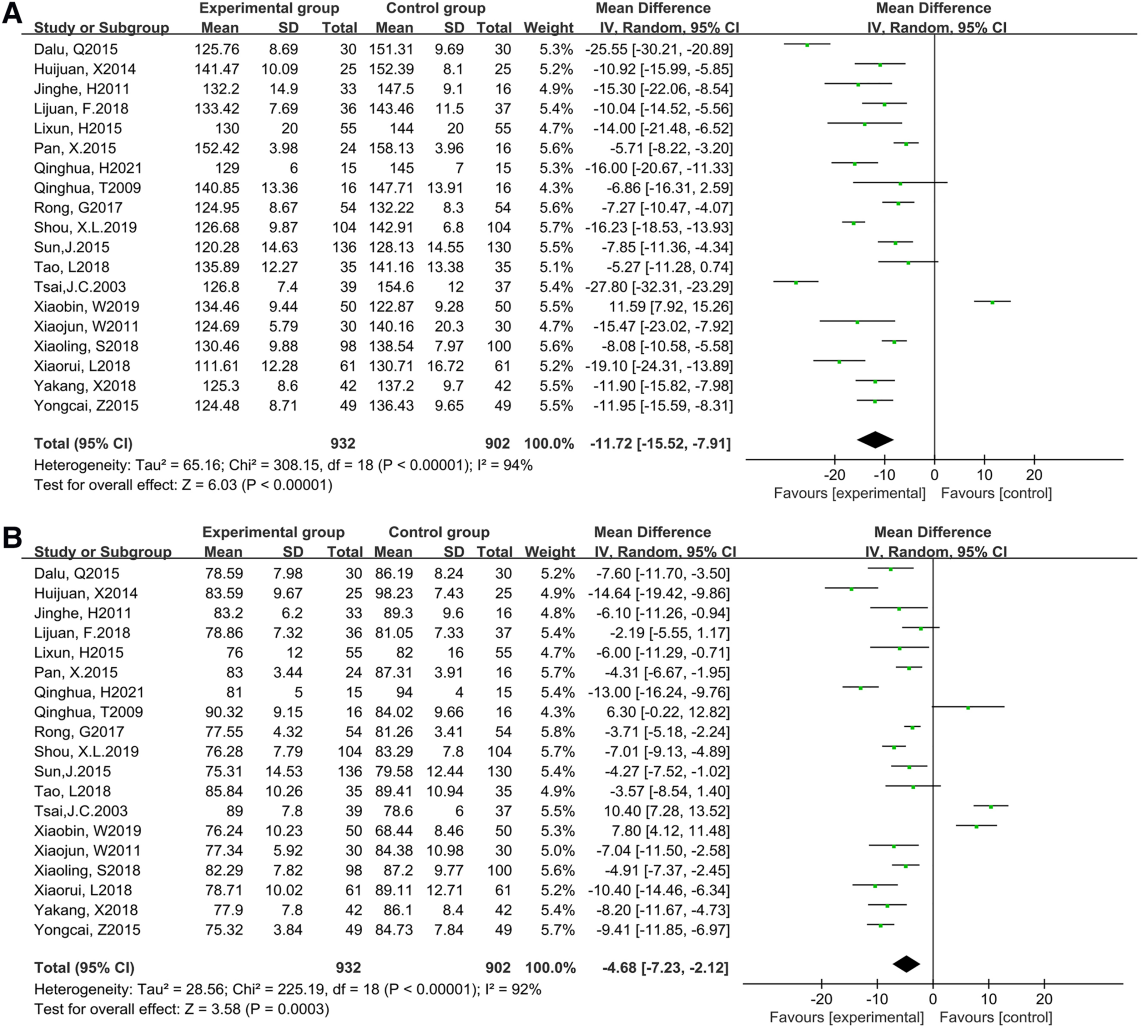
Forest plot of meta-analysis on the effect of Tai Chi exercise cycle on blood pressure (cycle≥12 weeks). **A**, systolic blood pressure (SBP); **B**, diastolic blood pressure (DBP).

Based on 19 studies (1,825 participants), we found there was heterogeneity in systolic blood pressure (*P* < 0.00001, *I*^2^ = 94%) [MD = −11.72, 95% CI (−15.52, −7.91), *P* < 0.00001] and diastolic blood pressure (*P* < 0.00001, *I*^2^ = 92%) [MD = −4.68, 95% CI (−7.23, −2.12), *P* < 0.00001] while the cycle was more than or equal to 12 weeks, which was analyzed by random effects model. The result was statistically significant, which meant that compared with the controls, Tai Chi exercises effectively reduced SBP and DBP. The results were displayed in Figure 4.

#### 3.4.2. The effect of Tai Chi exercise cycle on NO

**Figure 5:**
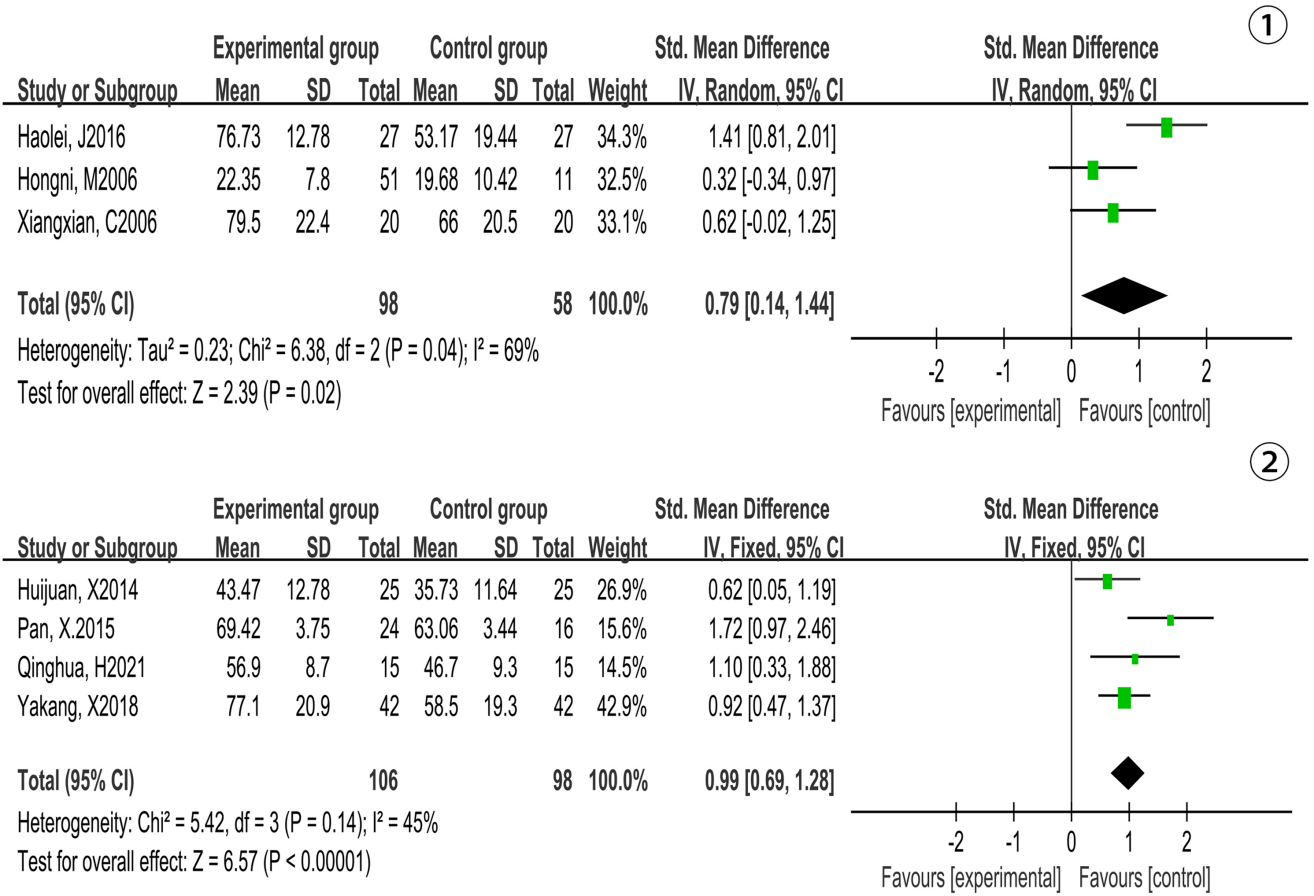
Forest plot of meta-analysis on the effect of Tai Chi exercise cycle on NO. Figure Notes:①, Cycle <12 weeks; ②, Cycle≥12 weeks.

Based on 3 studies (156 participants), we found there was heterogeneity in NO (*P* = 0.04, *I*^2^ = 69%) [MD = 0.79, 95% CI (0.14, 1.44), *P* = 0.02] while the cycle of Tai Chi exercise was less than 12 weeks, which was analyzed by random effects model. The result was not significant, which meant that compared with the control group, Tai Chi exercises failed to effectively increase the level of NO. The results were displayed in Figure 5-①.

Based on 4 studies (204 participants), we found there was low heterogeneity in NO (*P* = 0.14, *I*^2^ = 45%) [MD = 0.99, 95% CI (0.69, 1.28), *P* < 0.00001] while the cycle of Tai Chi exercise was more than or equal to 12 weeks, which was analyzed by fixed effects model. The result was significant, which meant that compared with the control group, Tai Chi exercise effectively increased the level of NO. The results were displayed in Figure 5-②.

#### 3.4.3. The effect of Tai Chi exercise cycle on blood lipid

**Figure 6:**
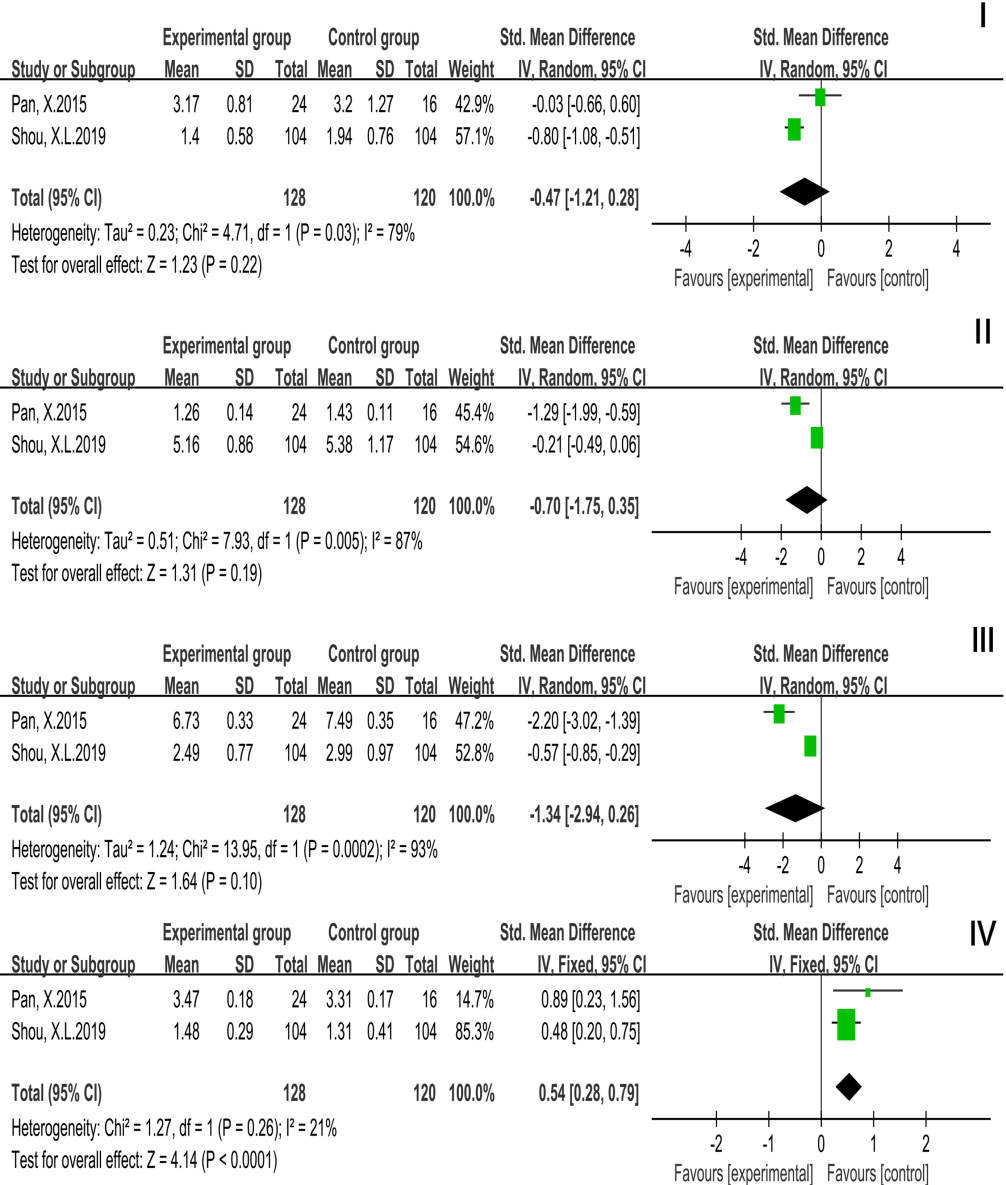
Forest plot of meta-analysis on the effect of Tai Chi exercise cycle on blood lipid related indexes (cycle <12 weeks). I, Total Cholesterol (TC); II, Triglyceride (TG); III, Low Density Lipoprotein (LDL-C); IV, High Density Lipoprotein (HDL-C).

Based on 2 studies (248 participants), we used random effects model analysis to draw conclusions: TC (*P* = 0.03, *I*^2^ = 79%) [SMD = −0.47, 95% CI (−1.21, 0.28), *P* = 0.22], TG (*P* = 0.005, *I*^2^ = 87%) [SMD = −0.70, 95% CI (−1.75, 0.35), *P* = 0.19], LDL-C (*P* = 0.0002, *I*^2^ = 93%) [SMD = −1.34, 95% CI (−2.94, 0.26), *P* = 0.10] and HDL-C (*P* = 0.26, *I*^2^ = 21%) [SMD = 0.54, 95% CI (0.28, 0.79), *P* < 0.0001]. The results were not significant, which meant that compared with the controls, TC, TG, and LDL-C were not improved but HDL-C was improved while Tai Chi exercise cycle was less than 12 weeks. The results were displayed in Figure 6.

**Figure 7:**
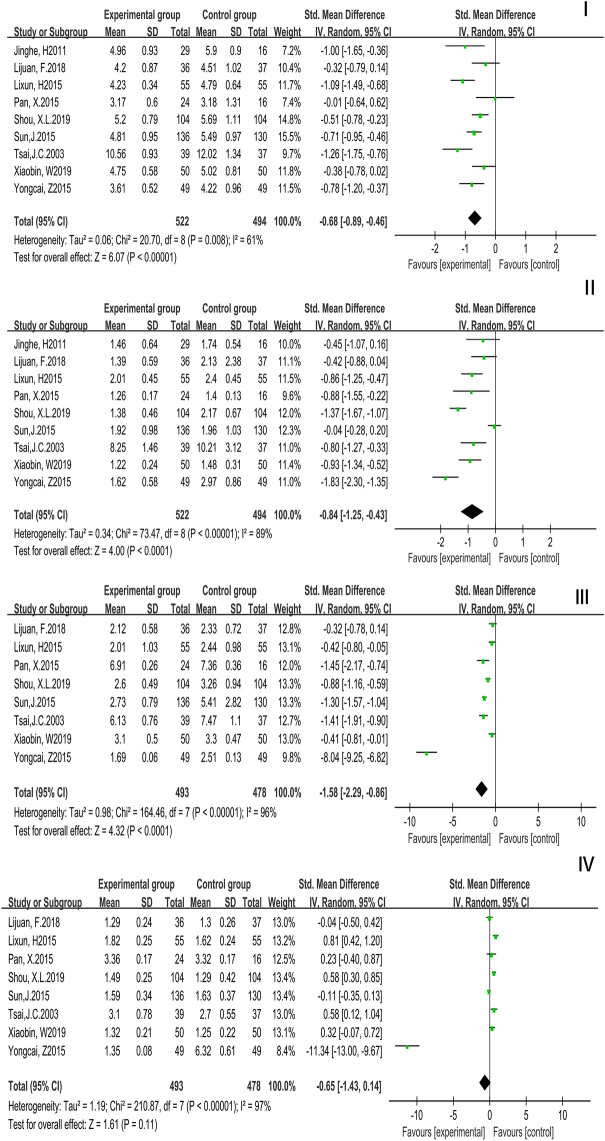
Forest plot of meta-analysis on the effect of Tai Chi exercise cycle on blood lipid related indexes (cycle ≥ 12 weeks). I, Total Cholesterol (TC); II, Triglyceride (TG); III, Low Density Lipoprotein (LDL-C); IV, High Density Lipoprotein (HDL-C).

A total of 9 studies with 1,016 patients reported TC and TG while Tai Chi exercise cycle was more than or equal to 12 weeks. A random effects model was used. TC (*P* = 0.03, *I*^2^ = 79%) (SMD = −0.68, 95% CI (−0.89, −0.46), TG (*P* = 0.005, *I*^2^ = 87%) [SMD = −0.84, 95% CI (−1.25, −0.43), *P* < 0.0001]. The results were statistically significantly different, which showed that compared with the controls, TC and TG were improved. A total of 8 studies with 971 patients reported LDL-C and HDL-C while Tai Chi exercise cycle was more than or equal to 12 weeks. A random effects model was used. LDL-C (*P* = 0.0002, *I*^2^ = 93%) [SMD = −1.58, 95% CI (−2.29, −0.86), *P* < 0.0001], HDL-C (*P* < 0.0001, *I*^2^ = 97%) [SMD = −0.65, 95% CI (−1.43, 0.14), *P* = 0.11]. The result of LDL-C was statistically significantly different while the result of HDL-C was not statistically significantly different, which showed that compared with the controls, LDL-C was improved but HDL-C was not. The results were displayed in Figure 7.

### 3.5. Subgroup analysis

Considering that variables such as exercise frequency and duration may alter the blood pressure and lipid metabolism of people with essential hypertension, a subgroup analysis was carried out. The impact of decreasing diastolic blood pressure level is substantial when the exercise frequency is less than 5 times per week, according to the subgroup analysis of the above components. When the exercise frequency is greater than 5 times per week, the effect on blood lipid metabolism is more noticeable. In terms of blood pressure, when exercise time is less than 60 min per day, the effect of decreasing blood pressure and enhancing blood lipid metabolism is more noticeable. Tables 2, 3 show the results.

**Table 2:** The subgroup analysis of blood pressure.

Index	Variable	Group	Sample size	Homogeneity test			Effect size and 95% CI	Two-tailed test	
				C^2^	*P*	I^2^		Z	*P*
SBP	Frequency	≥5/W	1,515	293.56	0.00	95%	−10.02 (−14.27, −5.77)	6.94	0.04
		<5/W	589	220.40	0.00	97%	−10.03 (−19.54, −0.53)	6.19	0.00
	Time	≥60 min/d	932	159.86	0.00	94%	−8.43 (−13.67, −3.18)	5.25	0.00
		<60 min/d	1,172	238.85	0.00	95%	−11.20 (−16.54, −5.86)	2.06	0.04
DBP	Frequency	≥5/W	1,515	163.08	0.00	91%	−5.51 (−8.08, −2.95)	5.56	0.00
		<5/W	589	252.38	0.00	97%	−0.88 (−6.97, 5.21)	1.90	0.78
	Time	≥60 min/d	932	102.91	0.02	91%	−3.97 (−7.40, −0.55)	4.20	0.02
		<60 min/d	1,172	217.83	0.01	94%	−4.13 (−8.17, −0.10)	0.93	0.04

**Table 3:** The subgroup analysis of lipid metabolism.

Index	Variable	Group	Sample size	Homogeneity test			Effect size and 95% CI	Two-tailed test	
				C^2^	*P*	I^2^		Z	*P*
TC	Frequency	≥5/W	248	0.24	0.62	0%	−0.19 (−0.45, 0.07)	1.47	0.14
		<5/W	347	16.88	0.00	82%	−0.63 (−1.06, −0.19)	2.22	0.00
	Time	≥60 min/d	113	0.44	0.51	0%	−0.23 (−0.60, 0.14)	1.23	0.22
		<60 min/d	482	19.72	0.00	85%	−0.59 (−1.01, −0.17)	2.76	0.00
TG	Frequency	≥5/W	248	13.21	0.00	92%	−0.35 (−0.71, 0.02)	1.87	0.06
		<5/W	347	55.79	0.00	95%	−1.00 (−1.79, −0.21)	2.48	0.01
	Time	≥60 min/d	113	1.98	0.16	49%	−0.23 (−0.60, 0.14)	4.45	0.00
		<60 min/d	482	56.66	0.00	95%	−0.86 (−1.37, −0.35)	3.28	0.00
LDL-C	Frequency	≥5/W	248	2.51	0.11	60%	−0.63 (−0.89, −0.38)	4.89	0.00
		<5/W	347	59.88	0.00	95%	−0.62 (−1.05, −0.19)	2.82	0.00
	Time	≥60 min/d	113	8.51	0.00	88%	−0.50 (−1.03, 0.04)	1.81	0.07
		<60 min/d	482	51.18	0.00	94%	−0.68 (−1.06, −0.31)	4.57	0.00
HDL-C	Frequency	≥5/W	248	0.02	0.89	0%	0.17 (0.09, 0.24)	4.47	0.00
		<5/W	347	2,858.42	0.00	100%	−1.13 (−3.25, 0.99)	1.04	0.30
	Time	≥60 min/d	113	4.39	0.04	77%	0.08 (−0.09, 0.24)	0.89	0.37
		<60 min/d	482	2,979.81	0.00	100%	−1.08 (−3.11, −0.94)	1.05	0.30

### 3.6. Publication bias

Begg's test was conducted to analyze publication bias for the outcome indicators of SBP and DBP. The results showed that there was both no significant publication bias while Tai Chi exercise cycle was less than 12 weeks or more than or equal to 12 weeks. SBP (*t* = −1.42, *P* = 0.173, *P* > 0.05), DBP (*t* = −0.62, *P* = 0.543, *P* > 0.05); SBP (*t* = 0.10, *P* = 0.927, *P* > 0.05), DBP (*t* = −0.47, *P* = 0.652, *P* > 0.05).The details were presented in Supplementary Figure S1–S4.

## 4. Discussion

Aerobic exercise may be an effective way to improve blood pressure in hypertensive patients (52). Aerobic exercise for 12 weeks resulted in a significant fall in blood pressure, which stabilized at 36 weeks (53). After 3 months of Tai Chi exercise, TC, TG, and LDL-C decreased and HDL-C increased in the hypertensive patients (40, 50). This meta-analysis indicated that when the Tai Chi exercise cycle was more than or equal to 12 weeks, the improvements in blood pressure and blood lipid metabolism were all significantly better than the situation when the exercise cycle was less than 12 weeks. Thus, for patients with essential hypertension, to achieve better blood pressure decreasing and blood lipid metabolism improving, the Tai Chi exercise cycle may need to be more than or equal to 12 weeks.

Normal persons and hypertension sufferers both experience a brief drop in blood pressure after vigorous activity. Exercise-induced hypotension is the term for this occurrence (54). Exercise-induced hypotension would last 18–24 h. As a result, exercising more than twice a week might have a superior antihypertensive impact (55). However, according to a study (56), there is no link between blood pressure drop and weekly exercise frequency, and blood pressure cannot be efficiently reduced by exercising more than 3 times a week. And aerobic exercise no less than 3 times a week is beneficial for the improvement of lipid metabolism in hypertensive patients (57). This meta-analysis indicated that when the Tai Chi exercise frequency was more than or equal to 5 times per week, the improvements in blood pressure and blood lipid metabolism were more obvious than when the frequency was less than 5 times per week. Thus, exercise frequency is positively correlated with the effects on blood pressure lowering and lipid metabolism improvement. The recommended frequency for patients with essential hypertension of Tai Chi exercise may be more than or equal to 5 times per week. For patients with hypertension combined with hyperlipidemia, efforts should probably also be made to guarantee an exercise frequency greater than or equal to 5 times per week.

The included studies focused on the 30 to 120 min of Tai Chi exercise time, which covered both preparation and completion time. Simply 20 min of effective exercise can have a significant antihypertensive impact (58). However, there are few research on the effect of exercise duration on blood lipids in hypertensive individuals. Studies have shown that there is not a positive correlation between Tai Chi exercise time and blood pressure lowering effect, and that the blood pressure lowering effect of Tai Chi exercise time of 120 min is not superior to that of exercise time of 91–120 min (55). This meta-analysis indicated that when the Tai Chi exercise time was less than 60 min per day, the improvements in blood pressure and blood lipid metabolism were better than when the time was more than or equal to 60 min per day. Therefore, the best exercise time of Tai Chi for patients with essential hypertension may be less than 60 min per day.

Hypertension is often accompanied by metabolic abnormalities such as hyperlipidemia, diabetes, obesity, and insulin resistance, and effective control of blood pressure can significantly reduce the occurrence of cardiovascular events (27, 59–61). Blood lipid metabolic disorders may cause damage to the vascular endothelium, which hypertrophies smooth muscle cells in the vessel wall, thereby causing structural changes in the vessels of large arteries, leading to an increase in blood pressure and the risk of cardiovascular disease (62, 63). Dyslipidemia induced damage to the renal microvasculature, is also one of the causes of hypertension (64). And the increase of blood pressure level with the consequent organism sympathetic excitability negatively affects lipid metabolism, exacerbating dyslipidemia. From this, a vicious circle of elevated blood pressure and dyslipidemia arises (65, 66). Long-term aerobic exercise improves lipoprotein protease activity and the ability of the skeletal muscle to utilize fatty acid supply, promoting lipid metabolism (67). Exercise may regulate the synthesis, transport, and catabolism of lipoprotein by regulating the activity of lecithin-cholesterol acyltransferase (LCAT), lipoprotein lipase (LPL), and hepatic-triglyceride lipase (HTGL). In addition, exercise-induced lipid utilization is regulated by lipolysis of TG within adipose tissue and muscle to deliver fatty acids (FA) to muscle and regulate FA transmembrane transport and mitochondrial metabolism in muscle cells for the purpose of improving lipid metabolism (68–70).

Tai Chi pays attention to three key elements, consisting of soul, power, and idea, and emphasizes on breath transmission and the use of thoughts (71). Tai Chi belongs to the group of low to moderate intensity aerobic exercise with low requirements for basic physical fitness and muscle strength, and is considered a type of physical exercise that is highly beneficial to health (72). When patients with essential hypertension perform Tai Chi, their muscles are relaxed, and nervous system function is modulated, thereby reflexively causing vasodilation for the purpose of reducing blood pressure (73). Vasodilator and constrictor factors secreted by vascular endothelium regulate the degree of vasodilation. And there is a direct relationship between high and low BP and vasodilation (12). Studies have shown that regular aerobic exercise in sedentary middle-aged and older adults can reduce CVD risk by preventing elastic artery stiffness and endothelial dysfunction through modulation of structural proteins, reduction of oxidative stress and inflammation, and restoration of nitric oxide bioavailability (74, 75).

## 5. Conclusion

According to Chinese Guidelines for the Prevention and Treatment of Hypertension, Tai Chi exercise is best when performed 3 times per week for 30–120 min per session in patients with essential hypertension. However, this meta-analysis indicated that a more than 12 weeks Tai Chi exercise cycle with less than 60 min each time and more than 5 times per week may be more beneficial in blood pressure reduction, NO level increasing and blood lipid metabolism improving in the comparison with the other exercise cycles. For patients with hypertension plus hyperlipidemia, exercise frequency of less than 5 times per week may be better. In the future, it is suggested that the standardized exercise prescription of Tai Chi should be explored more deeply to provide better evidence-based medical support for the use of Tai Chi in the clinic.

## 6. Limitation

1) The included studies were mainly published in Chinese. The differences in the studies may have impacts on the results of the meta-analysis.
2) Less follow-up was reported in the included studies. It is suggested that more attention should be paid to the prognostic situation in the future.
3) The implementation of blinding was not explicitly stated in part of the included studies. This may have contributed to a decrease in the level of evidence for the meta-analysis.
4) There were certain flaws in the design and implementation process of partially included studies, which may lead to bias in measurement and implementation.

## Data Availability

Publicly available datasets were analyzed in this study. This data can be found here: CNKI(https://www.cnki.net/). Wan Fang Databases (https://www.wanfangdata.com.cn/index.html). PubMed (https://pubmed.ncbi.nlm.nih.gov/). Web Of Science (https://clarivate.com/products/web-of-science/). Science Direct (https://www.sciencedirect.com/). Accession numbers can be found in the Supplementary Material.
