# Supplementary material for: Effects of the different Tai Chi exercise cycles on patients with essential hypertension: A systematic review and meta-analysis
Source: Front Cardiovasc Med. 2023 Mar 3;10:1016629. doi: 10.3389/fcvm.2023.1016629 (PMC10020615; doi:10.3389/fcvm.2023.1016629)
Supplement: Supplementary file 1 [file Datasheet1.pdf]

PRISMA 2020 Checklist

Section and Topic	Item #	Checklist item	Location where item is reported
**TITLE**			
Title	1	The report is identified as a meta-analysis	Page 1
**ABSTRACT**			
Abstract	2	To explore the Effects of the different Tai Chi exercise cycles on patients with essential hypertension:a systematic review and meta-analysis	Page 1
**INTRODUCTION**			
Rationale	3	Tai Chi is a prominent traditional Chinese martial art as well as a popular Chinese aerobic exercise. It's also recognized as a traditional type of rehabilitative training. Tai Chi is conducive to maintaining the stability of the vasomotor nerve, improving vascular compliance, reducing blood pressure, enhancing cardiorespiratory ability, and improving the quality of life.	Page 2
Objectives	4	This study aimed to systematically review the therapeutic effectiveness of the Tai Chi exercise cycle on blood pressure and cardiovascular risk factors of patients with essential hypertension.	Page 1
**METHODS**			
Eligibility criteria	5	Inclusion criteria: 1) The study design was a randomized controlled trial (RCT). 2) The research objects were patients with essential hypertension (unlimited sex, age, race, and nationality) according to the diagnostic criteria such as 1999/2005/2010/2016 Chinese guidelines for the management of hypertension, WHO-ISH (i.e., SBP ≥ 140 mmHg and/or DBP ≥ 90 mmHg). Patients with secondary hypertension and other severe cardio-cerebrovascular disease were required to be excluded. 3) The main intervention methods were Tai Chi exercise or other intervention methods combined with Tai Chi exercise. The intervention methods of the controls included pharmacotherapy, usual care, other exercise methods, or no treatment. 4) Raw data were complete and could be extracted directly or indirectly for analysis. 5) The publication language of the articles was Chinese or English. Exclusion criteria: 1) Duplicate published literature; 2) Inability to efficiently extract data and access the literature of original articles; 3) Animal studies or cross-sectional studies; 4) Experiments with nonclinical and nonintervention designs.	Page3
Information sources	6	A search of CNKI, VIP, CBM, PubMed, EBSCO, Embase, Cochrane Library, and Web of Science for RCTs on Tai Chi exercise on patients with essential hypertension published from the time of the databases established to December 2022 was conducted on December 13, 2022.	Page 3
Search strategy	7	We also searched to retrieve all potential relevant unpublished reported materials and conference proceedings referred to the topic. Search terms included “tai-ji”, “tai chi”, “chi, tai”, “tai ji quan”, “ji quan, tai”, “quan, tai ji”, “taiji”, “taijiquan”, “t'ai chi”, “tai chi chuan”, “hypertension”, “blood pressure, high”, “high blood pressure”, “hypertension, essential”, “essential hypertension”, “primary hypertension”, “human essential hypertension”, “idiopathic hypertension”. The full search strategies of each database were presented in Supplemental Material 1.	Page 3/ Supplemental Materials table1.
Selection process	8	The quality of the articles was assessed by two researchers, and any disagreements were resolved through discussion until a consensus was reached or after consultation with the third author.	Page 3
Data collection process	9	The three authors independently extracted the necessary data, and any disagreements were resolved through discussion until a consensus was reached or after consultation with the third author. The extraction of the relevant data from the included articles was then performed.	Page 3
Data items	10a	Outcome indicators included systolic blood pressure (SBP), diastolic blood pressure (DBP), total cholesterol (TC), triglycerides (TG), low-density lipoprotein cholesterol (LDL-C), high-density lipoprotein cholesterol (HDL-C) and serum nitric oxide (NO).	Page 3
	10b	Table 1 Basic Characteristics of Included Articles	Table 1
Study risk of bias assessment	11	RevMan 5.4 software was used to evaluate the included studies according to the Cochrane Manual of Systematic Reviews, and the literature quality grade was scored according to the Jadad scale (score 1-3 was considered as low quality, score 4-7 was considered as high quality).	Page 5/ Figure 2/ Supplemental Materials table 2
Effect measures	12	Statistical analysis was performed based on the meta-analysis software RevMan5.4 provided by the Cochrane collaboration. If the outcomes of all included literatures were continuous variables, mean difference (MD) and 95% Confidence Interval (CI) was used for statistics. P value and I^2 were used to quantitatively determine the heterogeneity among the included studies. $P \geq 0.10$ indicated that there was no heterogeneity among the studies, while $P<0.10$ indicated that there was heterogeneity among the studies. I^2 represents the level of heterogeneity between	Page 3

Section and Topic	Item #	Checklist item	Location where item is reported
		studies. If I^2<50%, it indicates that there is slight heterogeneity among studies, and the fixed effects model was used for analysis. If I^2≥50%, the study was heterogeneous, and the random effects model was used for analysis (Cochrane et al., 2021). The level of meta-analysis was set at α=0.05. Begg's test was used for publication bias analysis and sensitivity analysis for studies with ≥5 included studies by Stata12.0 software, and P<0.05 indicated significant difference.	
Synthesis methods	13a	Table 1 Basic Characteristics of Included Articles	Table 7
	13b	For the included articles, RevMan 5.4.1 software provided by Cochrane was used for the meta-analysis of all statistical analyses. Begg's test was used for publication bias analysis and sensitivity analysis by Stata12.0 software	Page 4
	13c	Outcome indicators included systolic blood pressure (SBP), diastolic blood pressure (DBP), total cholesterol (TC), triglycerides (TG), low-density lipoprotein cholesterol (LDL-C), high-density lipoprotein cholesterol (HDL-C) and serum nitric oxide (NO). MD values were used for effect evaluation, and 95% CI was calculated; the heterogeneity of the articles was evaluated through I^2. If the included articles had no statistical heterogeneity (P > 0.01, I^2 < 50%), the fixed effect model was used; if the included articles had statistical heterogeneity (P < 0.01, I^2 > 50%), the random effect model was used, and sensitivity analysis was performed to understand the source of the heterogeneity.	Page 3
Reporting bias assessment	14	Begg's test was conducted to analyze publication bias for the outcome indicators of SBP and DBP. The results showed that there was both no significant publication bias while Tai Chi exercise cycle was less than 12 weeks or more than or equal to 12 weeks. SBP (t=-1.42,P=0.173,P>0.05), DBP(t=-0.62,P=0.543,P>0.05); SBP (t=0.10,P=0.927,P>0.05), DBP(t=-0.47,P=0.652,P>0.05).The details were presented in Supplementary Figure S1-S4.	Page 16/ Supplemental Materials Figs S1-S4
Certainty assessment	15	Describe any methods used to assess certainty (or confidence) in the body of evidence for an outcome.	No
**RESULTS**			
Study selection	16a	Fig. 1 Process of Inclusion of Articles in Meta-analysis	Fig 1
Study characteristics	17	Cite each included study and present its characteristics.	Table 1
Risk of bias in studies	18	Present assessments of risk of bias for each included study.	Page 5/ Figure 2/ Supplemental Materials table 2
Results of individual studies	19	Cycle<12 weeks:<br>SBP (MD=-5.73，95%CI(-10.22，-1.25), P=0.01)<br>DBP (MD =-1.72，95%CI(-4.12，0.69), P=0.16)<br>NO(MD=0.79，95%CI(0.14,1.44), P=0.02)<br>TC(SMD=-0.47,95%CI (-1.21, 0.28),P=0.22)<br>TG (SMD=-0.70,95%CI (-1.75, 0.35),P=0.19)<br>LDL-C(SMD=-1.34,95%CI (-2.94, 0.26),P=0.10)<br>HDL-C(SMD=0.54,95%CI (0.28, 0.79),P＜0.0001)<br>Cycle≥12 weeks:<br>SBP (MD= -11.72，95%CI(-15.52,-7.91), P＜0.00001)<br>DBP (MD=-4.68，95%CI(-7.23,-2.12), P＜0.00001)<br>NO(MD=0.99，95%CI(0.69,1.28), P＜0.00001)<br>TC(SMD=-0.68,95%CI (-0.89, -0.46)<br>TG (SMD=-0.84,95%CI (-1.25, -0.43),P＜0.0001)	Page 10-16/ Fig 3-7/ Table 2-3 Supplemental Materials Figs S1-S4

PRISMA 2020 Checklist

Section and Topic	Item #	Checklist item	Location where item is reported
		LDL-C(SMD=-1.58,95%CI (-2.29, -0.86),P＜0.0001)<br>HDL-C(SMD=-0.65,95%CI (-1.43, 0.14),P=0.11)	
Results of syntheses	20a	For each synthesis, briefly summarise the characteristics and risk of bias among contributing studies.	Page 10-16/<br>Fig 3-7/<br>Table 2-3
	20b	Present results of all statistical syntheses conducted. If meta-analysis was done, present for each the summary estimate and its precision (e.g. confidence/credible interval) and measures of statistical heterogeneity. If comparing groups, describe the direction of the effect.	Page 10-16/<br>Fig 3-7/<br>Table 2-3
	20c	Present results of all investigations of possible causes of heterogeneity among study results.	Page 13
	20d	Present results of all sensitivity analyses conducted to assess the robustness of the synthesized results.	no
Reporting biases	21	The presence of publication bias was assessed using Egger's method.	Page 10-16/<br>Fig 3-7/<br>Table 2-3<br>Supplemental Materials Figs S1-S4
Certainty of evidence	22	Present assessments of certainty (or confidence) in the body of evidence for each outcome assessed.	Page 10-16/<br>Fig 3-7/<br>Table 2-3
**DISCUSSION**			
Discussion	23a	Provide a general interpretation of the results in the context of other evidence.	Page 16-18
	23b	Discuss any limitations of the evidence included in the review.	Page 16-18
	23c	Discuss any limitations of the review processes used.	Page 16-18
	23d	Discuss implications of the results for practice, policy, and future research.	Page 16
**OTHER INFORMATION**			
Registration and protocol	24	**We are in the process of registering.(PROSPERO)**	
Support	25	Describe sources of financial or non-financial support for the review, and the role of the funders or sponsors in the review.	Page 18-19
Competing interests	26	Declare any competing interests of review authors.	Page 18-19

From: Page MJ, McKenzie JE, Bossuyt PM, Boutron I, Hoffmann TC, Mulrow CD, et al. The PRISMA 2020 statement: an updated guideline for reporting systematic reviews. BMJ 2021;372:n71. doi: 10.1136/bmj.n71
For more information, visit: http://www.prisma-statement.org/
