# Supplementary material for: Effects of the different Tai Chi exercise cycles on patients with essential hypertension: A systematic review and meta-analysis
Source: Front Cardiovasc Med. 2023 Mar 3;10:1016629. doi: 10.3389/fcvm.2023.1016629 (PMC10020615; doi:10.3389/fcvm.2023.1016629)
Supplement: Supplementary file 2 [file Table1.docx]

| 纳入研究 | 随机序列的产生 | 随机化隐藏 | 盲法 | 撤出与退出 | 评分 | 等级 |
| --- | --- | --- | --- | --- | --- | --- |
| Tsai et al. (1) | 2 | 2 | 1 | 1 | 6 | H |
| Hongni and Peng (2) | 2 | 0 | 1 | 1 | 4 | H |
| Xiangxian and Qing (3) | 1 | 0 | 1 | 1 | 3 | L |
| Qinghua (4) | 2 | 1 | 1 | 1 | 5 | H |
| Jinghe et al. (5) | 0 | 0 | 1 | 1 | 2 | L |
| Xiaojun et al. (6) | 2 | 2 | 1 | 1 | 6 | H |
| Lo et al. (7) | 0 | 0 | 2 | 1 | 3 | L |
| Feng and Chunfeng (8) | 2 | 2 | 2 | 1 | 7 | H |
| Huijuan and Caiqin (9) | 2 | 2 | 1 | 0 | 5 | H |
| Dalu et al. (10) | 2 | 2 | 1 | 0 | 5 | H |
| Lixun and Jianquan (11) | 2 | 2 | 1 | 1 | 6 | H |
| Pan et al. (12) | 2 | 1 | 1 | 0 | 4 | H |
| Sun and Buys (13) | 2 | 1 | 1 | 1 | 5 | H |
| Yongcai et al. (14) | 2 | 1 | 1 | 1 | 5 | H |
| Chaoyang et al. (15) | 2 | 1 | 1 | 1 | 5 | H |
| Haolei and Jiajia (16) | 2 | 1 | 1 | 1 | 5 | H |
| Rong et al. (17) | 0 | 1 | 1 | 1 | 3 | L |
| Lijuan et al. (18) | 2 | 1 | 1 | 1 | 5 | H |
| Ma et al. (19) | 2 | 2 | 1 | 1 | 6 | H |
| Tao et al. (20) | 2 | 1 | 1 | 1 | 5 | H |
| Xiaoling et al. (21) | 2 | 1 | 1 | 1 | 5 | H |
| Xiaorui (22) | 0 | 1 | 1 | 1 | 3 | L |
| Yakang (23) | 2 | 1 | 1 | 1 | 5 | H |
| Shou et al. (24) | 2 | 1 | 1 | 1 | 5 | H |
| Xiaobin and Luping (25) | 2 | 1 | 1 | 1 | 5 | H |
| Qinghua et al. (26) | 2 | 1 | 1 | 1 | 5 | H |

1. Tsai, J.C., Wang, W.H., Chan, P., Lin, L.J., Wang, C.H., Tomlinson, B., et al. (2003). The beneficial effects of Tai Chi Chuan on blood pressure and lipid profile and anxiety status in a randomized controlled trial. *J Altern Complement Med* 9(5)**,** 747-754. doi: 10.1089/107555303322524599.
2. Hongni, M., and Peng, S. (2006). Effect of Tai Chi exer cise on blood pr essur e, plasma nitrogen　monoxidum and endothelin in hyper tensive patients. *Chinese Journal of Clinical Rehabilitation,* (48)**,** 65-67.
3. Xiangxian, C., and Qing, L.H. (2006). Effects of Taijiquan Exercise on Hypertension Patients’NO Consistency in Plasmathe Activity of RBC Na＋-K＋ ATPase and Ca2＋-Mg2＋ ATPase. Journal of Beijing Sport University (10), 1359-1361. doi: 10.19582/j.cnki.11-3785/g8.2006.10.023.
4. Qinghua, T. (2009). Effects of Traditional Sports on Clinical Symptom of AgedIntellectual Patients with Essential Hypertension. *Journal of Beijing Sport University* 32(02)**,** 67-69. doi: 10.19582/j.cnki.11-3785/g8.2009.02.017.
5. Jinghe, H., Li, Y., Zhen, C., and Guannan, L. (2011). Effect of Tai Ji Chuan intervention on primary hypertension. Chinese Journal of Rehabilitation Medicine 26(10), 968-971.
6. Xiaojun, W., Zhikun, J., and Ningning, Z. (2011). Effects of Taichi Exercises of Various Intensities on Essential Hypertension. *Journal of Shenyang Sport University* 30(04)**,** 82-85.
7. Lo, H.M., Yeh, C.Y., Chang, S.C., Sung, H.C., and Smith, G.D. (2012). A Tai Chi exercise programme improved exercise behaviour and reduced blood pressure in outpatients with hypertension. Int J Nurs Pract 18(6), 545-551. doi: 10.1111/ijn.12006.
8. Feng, S., and Chunfeng, S. (2014). Intervention effect of Tai Ji Chuan exercise on senile hypertension. *Chinese journal of gerontology* 34(24)**,** 6862-6864.
9. Huijuan, X., and Caiqin, B. (2014). Gaseous Signal Molecular Mechanism of Taijiquan's Intervention in Aged　Essential Hypertension. *Journal of Wuhan Institute of Physical Education* 48(02)**,** 51-54+63. doi: 10.15930/j.cnki.wtxb.2014.02.001.
10. Dalu, Q., Yongming, L., and Xinghui, Y. (2015). Influence of Taijiquan on Eight Patients with Hypertension Blood Pressure Levels. *SICHUAN SPORTS SCIENCE* 34(05)**,** 24-26+39. doi: 10.13932/j.cnki.sctykx.2015.05.07.
11. Lixun, H., and Jianquan, Y. (2015). Effects of vigorous walk combined with Taijiquan on blood glucose and lipid, blood pressure in elderly hypertensive patients. *Hainan Med J* 26(14)**,** 2053-2055.
12. Pan, X., Zhang, Y., and Tao, S. (2015). Effects of Tai Chi exercise on blood pressure and plasma levels of nitric oxide, carbon monoxide and hydrogen sulfide in real-world patients with essential hypertension. Clin Exp Hypertens 37(1), 8-14. doi: 10.3109/10641963.2014.881838.
13. Sun, J., and Buys, N. (2015). Community-Based Mind-Body Meditative Tai Chi Program and Its Effects on Improvement of Blood Pressure, Weight, Renal Function, Serum Lipoprotein, and Quality of Life in Chinese Adults With Hypertension. *Am J Cardiol* 116(7)**,** 1076-1081. doi: 10.1016/j.amjcard.2015.07.012.
14. Yongcai, Z., Liang, C., and Jianquan, Y. (2015). Taijiquan Systematic Reviews of Primary Effects on Blood Pressureand Ouality of Life of Patients with Hypertension. Journal of liaoning university of TCM 17(04), 143-146. doi: 10.13194/j.issn.1673-842x.2015.04.050.
15. Chaoyang, L., Liang, W., and Ruiling, L. (2016). The influence of exercise treatment on typeⅠhypertension of elders. *Journal of Henan University( Medical Science)* 35(04)**,** 287-289. doi: 10.15991/j.cnki.41-1361/r.2016.04.017.
16. Haolei, J., and Jiajia, P. (2016). Effects of Taijiquan on 24-hour Dynamic Blood Pressure and Vascular Function in Patients with Primary Mild Hypertension. *Chin J Sports Med* 35(03)**,** 224-227. doi: 10.16038/j.1000-6710.2016.03.004.
17. Rong, G., Xiuqin, H., Fengling, W., and Juan, W. (2017). Effect of Tai Ji Chuan exercise combined with newman system health care model on blood pressure and quality of life of elderly patients with hypertension. *Chin J Ctrl Endem Dis* 32(07)**,** 823+825.
18. Lijuan, F., Li, G., DaiLiang, Z., Zhongchun, L., Mengxiao, L., and Rongjiang, J. (2018). Clinical evaluation of 24-type Tai Ji Chuan on antihypertensive effect and blood lipid level in elderly patients with primary hypertension. *Chin J Convalescent Med* 27(10)**,** 1009-1013. doi: 10.13517/j.cnki.ccm.2018.10.001.
19. Ma, C., Zhou, W., Tang, Q., and Huang, S. (2018). The impact of group-based Tai chi on health-status outcomes among community-dwelling older adults with hypertension. *Heart Lung* 47(4)**,** 337-344. doi: 10.1016/j.hrtlng.2018.04.007.
20. Tao, L., Qidong, H., and Weizhong, L. (2018). Effect of Tai Ji Chuan exercise on blood pressure, hemorheology and long-term quality of life in elderly patients with hypertension. *Chinese journal of gerontology* 38(06)**,** 1396-1398.
21. Xiaoling, S., Wang, L., liyue, Z., Hua, R., Wanzhen, W., Ping, X.S., et al. (2018). Effect of Tai Ji Chuan exercise on emotion and heart rate variability in patients with hypertension. *Modern chinese doctor* 56(29)**,** 95-99.
22. Xiaorui, L. (2018). Effect of Tai Ji Chuan exercise combined with comprehensive nursing intervention on blood pressure control and quality of life of patients with essential hypertension. *Chinese journal of convalescent medicine* 27(01)**,** 19-21. doi: 10.13517/j.cnki.ccm.2018.01.007.
23. Yakang, X. (2018). Effects of Eight-Tai Ji Chuan Exercise on Blood Pressure Level, Vascular Endothelial Function and Quality of Life of Patients with Essential Hypertension. *chinese journal of gerontology* 38(10)**,** 2403-2405.
24. Shou, X.L., Wang, L., Jin, X.Q., Zhu, L.Y., Ren, A.H., and Wang, Q.N. (2019). Effect of T'ai Chi Exercise on Hypertension in Young and Middle-Aged In-Service Staff. *J Altern Complement Med* 25(1)**,** 73-78. doi: 10.1089/acm.2018.0011.
25. Xiaobin, W., and Luping, Y. (2019). Effect of 24-type simplified Tai Ji Chuan on primary hypertension with mild anxiety in the elderly. *Fujian Journal of TCM* 50(04)**,** 73-75. doi: 10.13260/j.cnki.jfjtcm.011881.
26. Qinghua, H., Youling, G., Xiaoli, L., Huan, Z., and Jiewen, X. (2021). Effects of 12-week Tai Chi exercise on the microvascular reactivityof the middle-aged and elderly patients with mild hypertensionand its mechanism. *Chin J Appl Physiol* 37(06)**,** 683-687.
