# Supplementary material for: Effects of the different Tai Chi exercise cycles on patients with essential hypertension: A systematic review and meta-analysis
Source: Front Cardiovasc Med. 2023 Mar 3;10:1016629. doi: 10.3389/fcvm.2023.1016629 (PMC10020615; doi:10.3389/fcvm.2023.1016629)
Supplement: Supplementary file 3 [file Table2.docx]

Supplementary Material

Supplementary Figures and Tables

Supplementary Figures


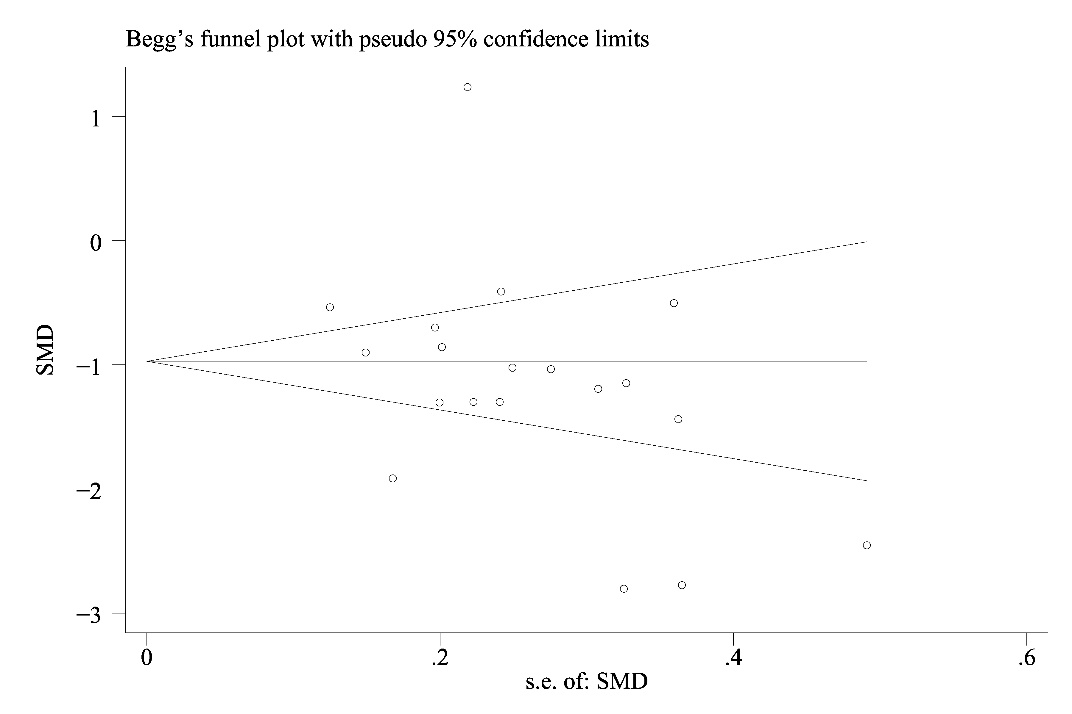


Fig S1 Systolic pressure publication bias map for inclusion in the literature(duration ≥12 weeks)
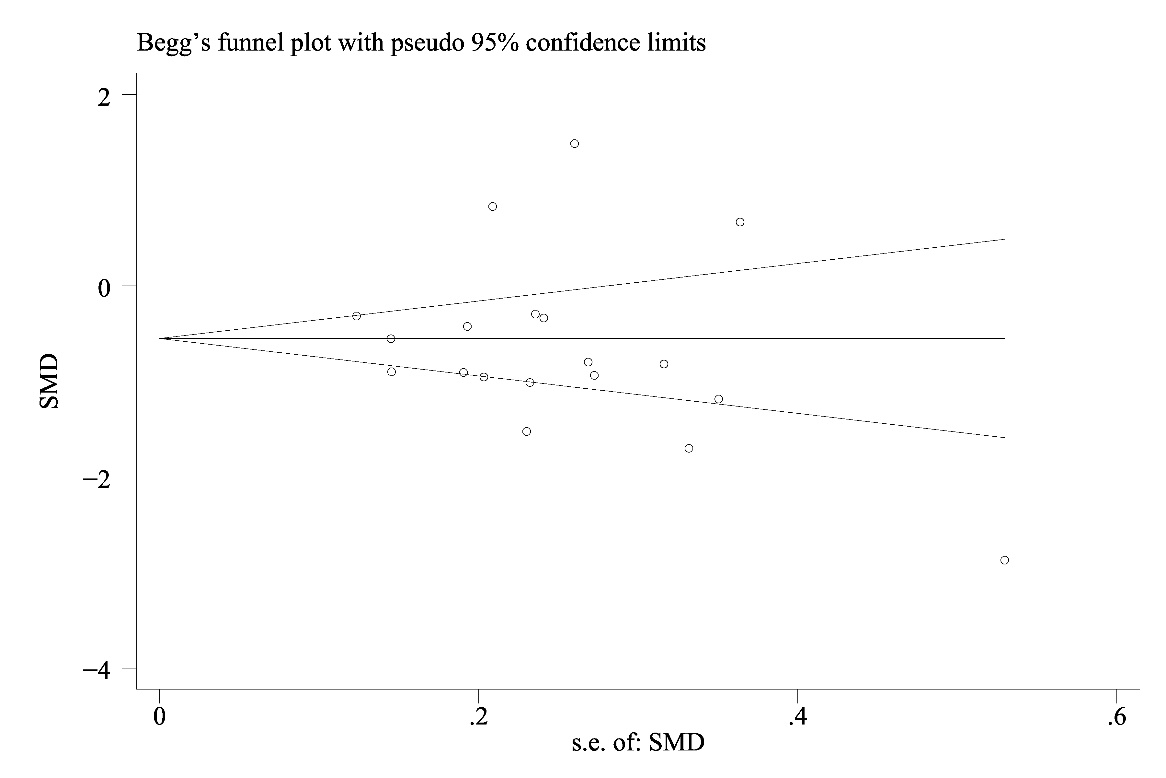


Fig S2 Diastolic pressure publication bias map for inclusion in the literature(duration ≥12 weeks)
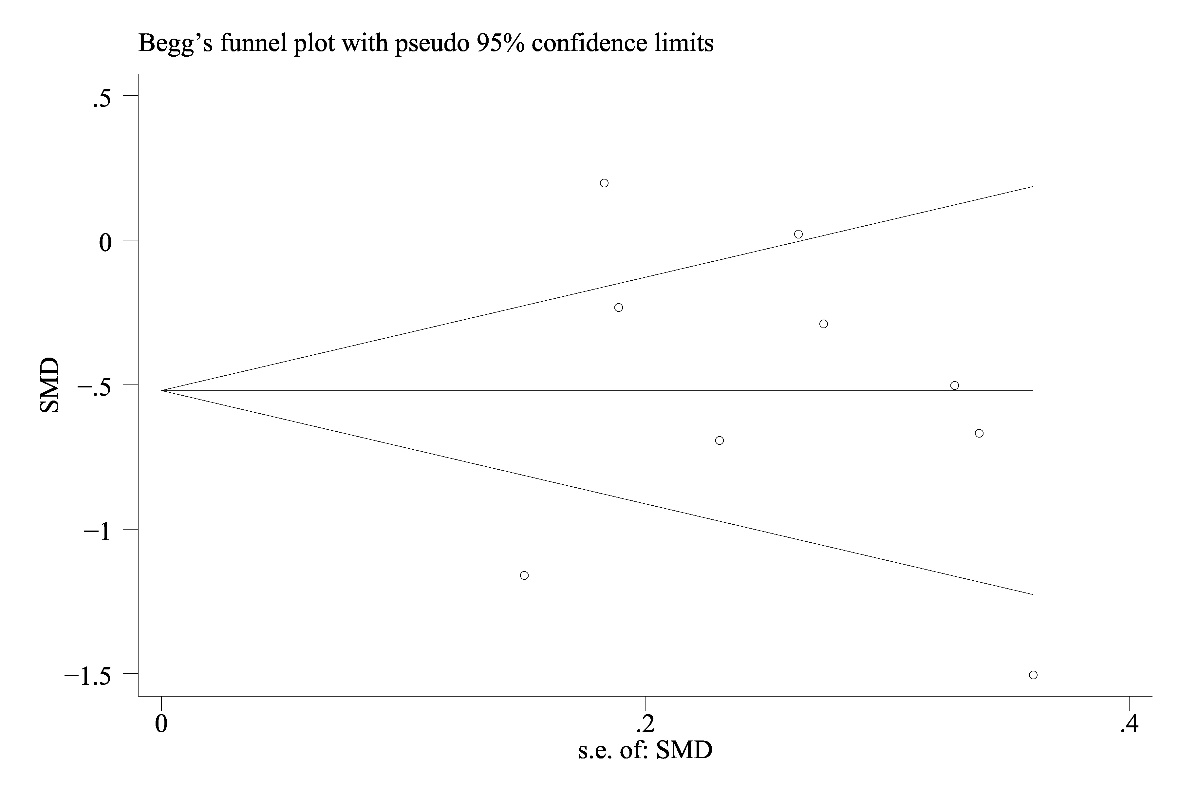


Fig S3 Systolic pressure publication bias map for inclusion in the literature(duration＜12 weeks)
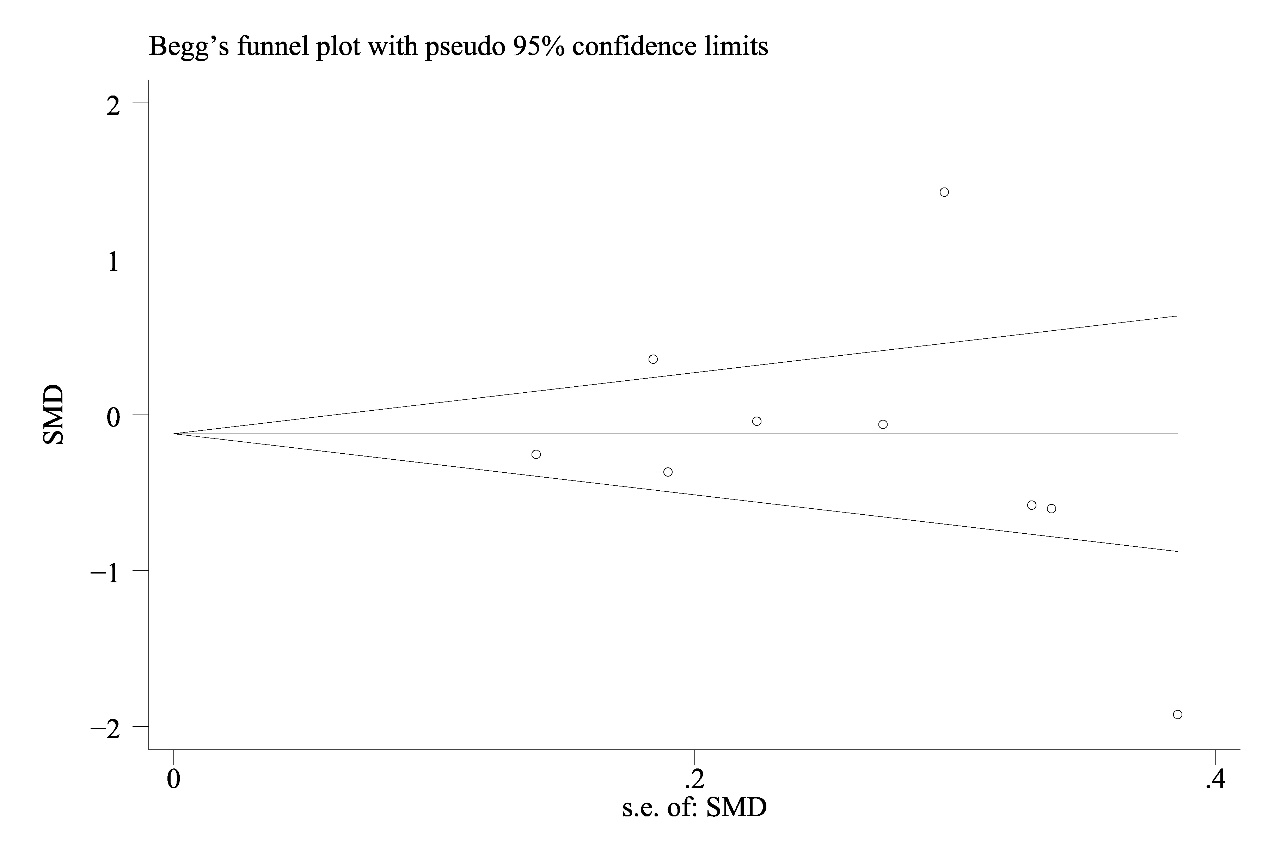


Fig S4 Diastolic pressure publication bias map for inclusion in the literature(duration＜12 weeks)

Supplementary Tables

Supplementary Table 1.Search strategy for each database

| Search engine | Search query | Date of search |
| --- | --- | --- |
| CNKI/VIP/ CBM | #1 主题 (Topic) =太极(Tai-ji, Tai Chi) OR 太极拳(Taijiquan, Tai Chi Chuan)  #2 主题 (Topic) =高血压(High Blood Pressure, hypertension) OR 原发性高血压(Primary Hypertension)  #3 #1 AND #2 | 13- December-22 |
| Pubmed | #1 Tai Chi [MeSH Terms]  #2 Tai-ji [Text Word]  #3 Chi, Tai [Text Word]  #4 Tai Ji Quan [Text Word]  #5 Ji Quan, Tai [Text Word]  #6 Quan, Tai Ji [Text Word]  #7 Tai Chi Chuan [Text Word]  #8 #1 OR #2 OR #3 OR #4 OR #5 OR #6 OR #7  #9 hypertension [MeSH Terms]  #10 High Blood Pressure [Text Word]  #11 Primary Hypertension [Text Word]  #12 Hypertension, Primary [Text Word]  #13 Blood Pressure, High [Text Word]  #14 Primary Hypertensions [Text Word]  #15 Hypertension, Essential [Text Word]  #16 Essential hypertension [Text Word]  #17 #9 OR #10 OR #11 OR #12 OR #13 OR #14 OR #15 OR #16  #18 #88AND #17 | 13- December-22 |
| Web of science | #1 TS = (‘Tai Chi’ OR ‘Tai-ji’ OR ‘Chi, Tai’ OR ‘Tai Ji Quan OR ‘Ji Quan, Tai ’ OR ‘Quan, Tai Ji ’ OR ‘Tai Chi Chuan’)  #2 TS = (‘hypertension’ OR ‘High Blood Pressure’ OR ‘Primary Hypertension’ OR ‘Hypertension, Primary’ OR ‘Blood Pressure, High’ OR ‘Primary Hypertensions’ OR ‘Hypertension, Essential’ OR ‘Essential hypertension’)  #3 #1 AND #2  Databases = SCI-EXPANDED, SSCI, A&HCI, CPCI-S, CPCI-SSH, ESCI | 13- December-22 |
| Embase | #1 ‘Tai Chi’ OR ‘Tai-ji’ OR ‘Chi, Tai’ OR ‘Tai Ji Quan OR ‘Ji Quan, Tai ’ OR ‘Quan, Tai Ji ’ OR ‘Tai Chi Chuan’  #2 ‘hypertension’ OR ‘High Blood Pressure’ OR ‘Primary Hypertension’ OR ‘Hypertension, Primary’ OR ‘Blood Pressure, High’ OR ‘Primary Hypertensions’ OR ‘Hypertension, Essential’ OR ‘Essential hypertension’  #3 #1 AND #2 | 13- December-22 |
| EBSCO | S1 Tai Chi’ OR ‘Tai-ji’ OR ‘Chi, Tai’ OR ‘Tai Ji Quan OR ‘Ji Quan, Tai ’ OR ‘Quan, Tai Ji ’ OR ‘Tai Chi Chuan’)  S2 ‘hypertension’ OR ‘High Blood Pressure’ OR ‘Primary Hypertension’ OR ‘Hypertension, Primary’ OR ‘Blood Pressure, High’ OR ‘Primary Hypertensions’ OR ‘Hypertension, Essential’ OR ‘Essential hypertension’  #3 #1 AND #2 | 13- December-22 |
| Cochrane Library | #1 ‘Tai Chi’ OR ‘Tai-ji’ OR ‘Chi, Tai’ OR ‘Tai Ji Quan OR ‘Ji Quan, Tai ’ OR ‘Quan, Tai Ji ’ OR ‘Tai Chi Chuan’  #2 ‘hypertension’ OR ‘High Blood Pressure’ OR ‘Primary Hypertension’ OR ‘Hypertension, Primary’ OR ‘Blood Pressure, High’ OR ‘Primary Hypertensions’ OR ‘Hypertension, Essential’ OR ‘Essential hypertension’  #3 #1 AND #2 | 13- December-22 |

Table note: CNKI:China National Knowledge Infrastructure; VIP: VIP database; CBM: China Biology Medicine disc.

Supplementary Table 2. Jadad scale score of included literature

| Reference | Generation of random sequences | randomization concealment | blind method | Withdrawal and withdrawal | score | level |
| --- | --- | --- | --- | --- | --- | --- |
| Tsai et al., 2003 | 2 | 2 | 1 | 1 | 6 | H |
| Hongni and Peng, 2006 | 2 | 0 | 1 | 1 | 4 | H |
| Xiangxian and qing, 2006 | 1 | 0 | 1 | 1 | 3 | L |
| Qinghua, 2009 | 2 | 1 | 1 | 1 | 5 | H |
| Jinghe et al., 2011 | 0 | 0 | 1 | 1 | 2 | L |
| Xiaojun et al., 2011 | 2 | 2 | 1 | 1 | 6 | H |
| Lo et al., 2012 | 0 | 0 | 2 | 1 | 3 | L |
| Feng and Chunfeng, 2014 | 2 | 2 | 2 | 1 | 7 | H |
| Huijuan and Caiqin, 2014 | 2 | 2 | 1 | 0 | 5 | H |
| Dalu et al., 2015 | 2 | 2 | 1 | 0 | 5 | H |
| Lixun and Jianquan, 2015 | 2 | 2 | 1 | 1 | 6 | H |
| Pan et al., 2015 | 2 | 1 | 1 | 0 | 4 | H |
| Sun and Buys, 2015 | 2 | 1 | 1 | 1 | 5 | H |
| Yongcai et al., 2015 | 2 | 1 | 1 | 1 | 5 | H |
| Chaoyang et al., 2016 | 2 | 1 | 1 | 1 | 5 | H |
| Haolei and Jiajia, 2016 | 2 | 1 | 1 | 1 | 5 | H |
| Rong et al., 2017 | 0 | 1 | 1 | 1 | 3 | L |
| Lijuan et al., 2018 | 2 | 1 | 1 | 1 | 5 | H |
| Ma et al., 2018 | 2 | 2 | 1 | 1 | 6 | H |
| Tao et al., 2018 | 2 | 1 | 1 | 1 | 5 | H |
| Xiaoling et al., 2018 | 2 | 1 | 1 | 1 | 5 | H |
| Xiaorui, 2018 | 0 | 1 | 1 | 1 | 3 | L |
| Yakang, 2018 | 2 | 1 | 1 | 1 | 5 | H |
| Shou et al., 2019 | 2 | 1 | 1 | 1 | 5 | H |
| Xiaobin and Luping, 2019 | 2 | 1 | 1 | 1 | 5 | H |
| Qinghua et al., 2021 | 2 | 1 | 1 | 1 | 5 | H |

Table note: H: High quality; L:Low quality.
